# Neoadjuvant Chemotherapy for Stage II Rectal Cancer Diagnosed in the Second Trimester of Pregnancy: A Case Report

**DOI:** 10.70352/scrj.cr.25-0208

**Published:** 2025-06-25

**Authors:** Haruka Kubo, Daisuke Yamamoto, Shunsuke Takenaka, Hiroyuki Tanaka, Ryosuke Machi, Kazuyoshi Mitta, Hiroshi Saito, Kenta Doden, Yusuke Sakimura, Hiroto Saito, Toshikatsu Tsuji, Hideki Moriyama, Jun Kinoshita, Noriyuki Inaki

**Affiliations:** Department of Gastrointestinal Surgery, Kanazawa University, Kanazawa, Ishikawa, Japan

**Keywords:** colorectal cancer, pregnancy, neoadjuvant chemotherapy, second trimester, case report

## Abstract

**INTRODUCTION:**

The onset of colorectal cancer during pregnancy is rare, and no standard treatment has been established. In this report, we present the case of a woman with clinical stage II rectal cancer diagnosed in the second trimester, in which neoadjuvant chemotherapy was administered, followed by delivery once fetal development was sufficiently advanced, and surgery was performed afterward.

**CASE PRESENTATION:**

The patient was a 36-year-old woman at 22 weeks of gestation. Sigmoidoscopy was performed for hematochezia, which revealed a semicircular type 2 tumor in the rectum. A biopsy confirmed the presence of adenocarcinoma. A thorough systemic examination revealed no lymph nodes or distant metastases. After discussing the risks and benefits with the patient, her family, a pediatrician, and an obstetrician, we decided to administer neoadjuvant chemotherapy. The plan was to deliver the fetus after it had adequately developed and then perform radical surgery for rectal cancer after delivery. Neoadjuvant chemotherapy comprised 4 courses of the modified FOLFOX6 regimen, including leucovorin calcium (folinic acid), fluorouracil, and oxaliplatin. The patient had a vaginal delivery at 35 weeks and 5 days of gestation, 23 days after the last chemotherapy dose. The newborn was healthy with no congenital anomalies. On the 27th day after delivery, a robot-assisted low anterior resection of the rectum was performed. The pathological findings revealed rectal cancer located above the peritoneal reflection, ypT2N0M0, and ypStage I. The patient recovered well and was discharged 12 days after surgery. At the time of writing, both the mother and child are doing well, with no evidence of recurrence 6 months after surgery.

**CONCLUSIONS:**

In cases of colorectal cancer during pregnancy, it is important to select a treatment plan that considers the site and stage of the tumor, number of weeks of pregnancy, and conditions of the fetus and mother. Even in cases of clinical stage II colorectal cancer diagnosed during the second trimester, where immediate surgery is not feasible, neoadjuvant chemotherapy can be considered a viable treatment option.

## Abbreviation

SGA
: small for gestational age

## INTRODUCTION

Colorectal cancer diagnosed during pregnancy is rare, occurring in approximately 2 cases per 100000.^1)^ Therefore, no standard treatment has been established for pregnant individuals with colorectal cancer. Moreover, controversy remains over the timing of surgery, whether neoadjuvant chemotherapy should be used, and the timing of delivery.

In this report, we present a case of clinical stage II rectal cancer diagnosed in the second trimester, in which neoadjuvant chemotherapy was administered, followed by delivery once the fetus had developed sufficiently, and then surgery was performed.

## CASE PRESENTATION

**Fig. 1:**
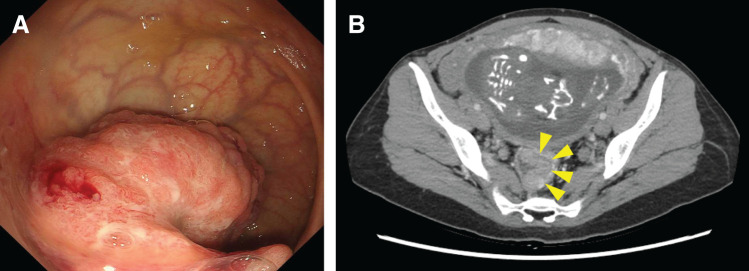
Pretreatment examination findings. (**A**) Sigmoidoscopy images revealing a semicircular type 2 tumor in the rectum above the peritoneal reflection. A biopsy confirmed a diagnosis of adenocarcinoma (group 5). (**B**) Enhanced computed tomography showing thickening of the rectal wall above the peritoneal reflection (arrowheads), with no evidence of invasion into surrounding tissues, lymph node metastasis, or distant metastasis.

The patient was a 36-year-old woman at 22 weeks of gestation. She was referred to the Department of Gastroenterology at our hospital with the chief complaint of hematochezia. Sigmoidoscopy revealed a semicircular type 2 tumor in the rectum above the peritoneal reflection. A biopsy confirmed the presence of adenocarcinoma in group 5 (**Fig. 1A**). Enhanced CT revealed rectal wall thickening above the peritoneal reflection; however, no invasion of the surrounding area, lymph node metastasis, or distant metastasis was noted (**Fig. 1B**). Accordingly, the pretreatment diagnosis was rectal cancer above the peritoneal reflection, cT3N0M0, cStage Ⅱa. The patient was referred to our department for treatment in the same month. We presented 2 treatment options: the 1st was to wait until the 28th week of pregnancy, when the fetus could be safely delivered via cesarean section, followed by a period for uterine involution before performing curative resection of the rectal cancer. The second option was to initiate neoadjuvant chemotherapy, allow the fetus to develop further, and then proceed with curative resection following delivery and uterine involution. The patient chose to proceed with the 2nd option. Therefore, we opted for neoadjuvant chemotherapy with FOLFOX after thorough discussions with the patient, an obstetrician-gynecologist, a pediatrician, and the medical safety manager.

Four courses of chemotherapy were administered without any adverse events. Twenty-three days after the final administration, the patient was induced at 35 weeks and 5 days of pregnancy. She gave birth to a baby girl weighing 2386 g via vaginal delivery. Although the baby was a low-weight premature infant, she was appropriate for gestational age and exhibited no obvious abnormalities or malformations.

**Fig. 2:**
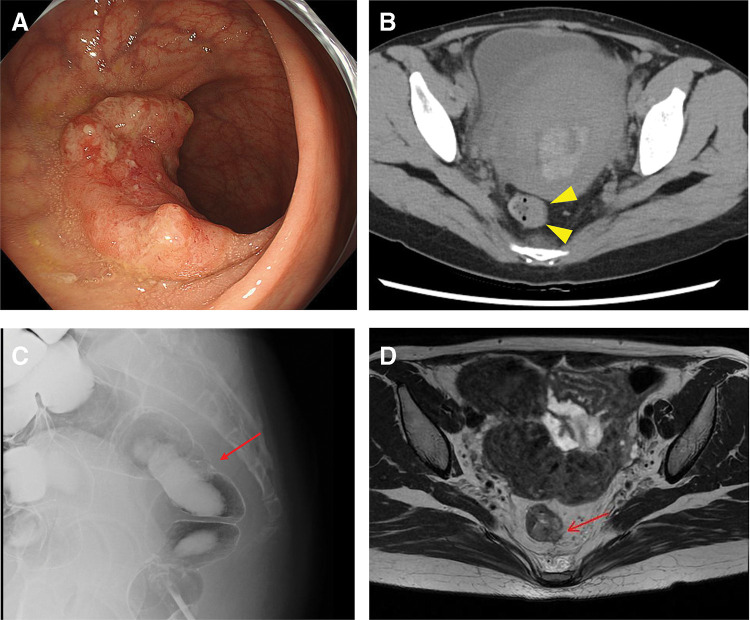
Neoadjuvant chemotherapy and post-delivery findings. (**A**) Colonoscopy images demonstrating shrinkage of the primary tumor compared to pretreatment findings. (**B**) Computed tomography confirming reduction in the size of the primary tumor (arrowheads), with no new lesions identified. (**C**) Barium enema revealing an irregularly elevated lesion with wall deformity in the upper rectum (arrow). (**D**) Contrast-enhanced magnetic resonance imaging indicating partial rupture and irregularity of the low-intensity muscularis propria (arrow), suggesting a T3 invasion depth.

**Fig. 3:**
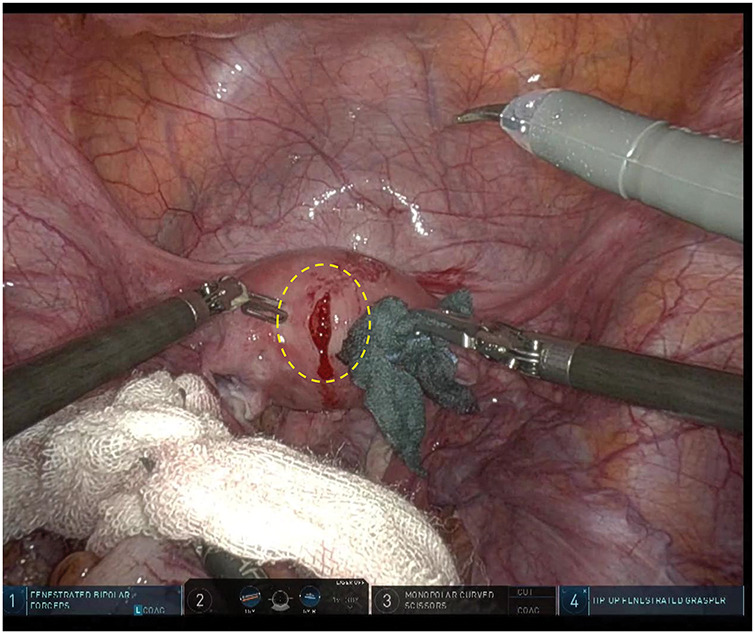
Intraoperative findings. Robot-assisted low anterior resection of the rectum with D3 lymph node dissection was performed. The uterus had contracted postpartum, allowing standard rectal cancer surgery. However, during pelvic manipulation, lifting of the uterus caused a tear due to its puerperal state (dotted circle), which was repaired by an obstetrician-gynecologist via suturing.

Colonoscopy and CT after chemotherapy and delivery indicated that the primary lesion had shrunk from the previous examination, and no new lesions developed (**Fig. 2A** and **2B**). A barium enema examination revealed an irregularly elevated lesion with wall deformation in the rectum above the peritoneal reflection (**Fig. 2C**). Contrast-enhanced magnetic resonance imaging indicated low-intensity signals, indicating the muscularis propria was partially ruptured and irregular, suggesting a T3 invasion depth (**Fig. 2D**). Accordingly, the effect of neoadjuvant chemotherapy was judged to be a stable disease. Therefore, robot-assisted low anterior resection of the rectum and D3 lymph node dissection were performed on the 27th day after delivery for rectal cancer above the peritoneal reflection, ycStage IIa. The operation lasted 4 h 35 min, with a blood loss volume of 10 mL. As the uterus had shrunk, visualization of the pelvis was not difficult, and no special techniques, such as transanal total mesorectal excision, were required; standard rectal cancer surgery was feasible, and reconstruction using end-to-end anastomosis was performed. During the pelvic procedure, the uterus was inadvertently ruptured by the puerperal uterus (**Fig. 3**), prompting consultation with an obstetrician-gynecologist for repair.

**Fig. 4:**
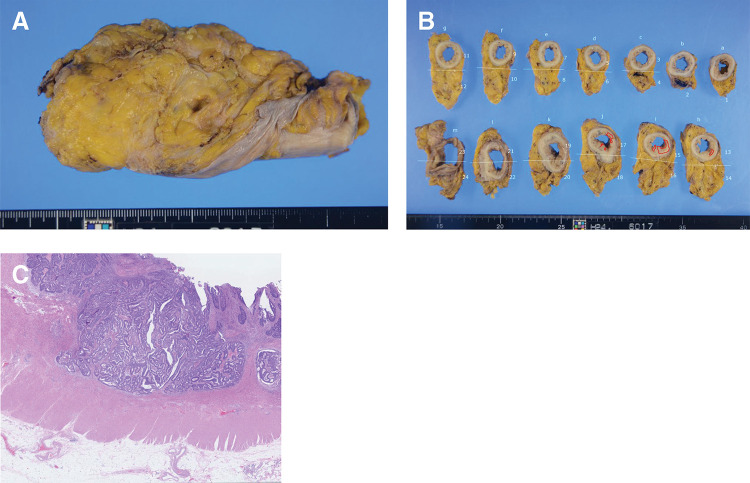
Surgical specimen and loupe image. (**A** and **B**) Gross specimens from low anterior resection of the rectum (the right side represents the oral side, the left side is the anal side, the top is the dorsal side, and the bottom is the ventral side), with gross appearance presented in (**B**). The areas surrounded by red lines indicate the location of the cancer. (**C**) Loupe image of hematoxylin and eosin staining. The postoperative diagnosis was ypT2N0M0, stage I.

The postoperative course progressed smoothly, and the patient was discharged on the 12th postoperative day without complications. The postoperative diagnosis was ypT2N0M0, ypStage I (**Fig. 4A**–**4C**), with proficient mismatch repair and negative RAS/BRAF mutation status. Although this staging indicated no lymph node involvement, we discussed the possibility of pre-existing lymph node metastasis with the patient. At the patient’s strong request, 8 courses of adjuvant FOLFOX chemotherapy were administered and completed. No recurrence has been observed as of the most recent examination. Six months have now passed since the surgery, and both the mother and child are doing well.

## DISCUSSION

The incidence of malignant tumors during pregnancy ranges between 0.07% and 0.1%.^2)^ The most common cancers diagnosed during pregnancy are breast cancer, melanoma, and cervical cancer, accounting for more than 60% of all cases. Cancers that are rarely diagnosed during pregnancy include brain and nervous system tumors (9.3%), lymphoma (6.8%), thyroid cancer (3.8%), and colorectal cancer (2.8%).^3)^ However, with the rising age of childbearing, the incidence of colorectal cancer during pregnancy may increase, necessitating proactive management strategies.

Colorectal cancer typically presents with symptoms such as changes in bowel habits (e.g., diarrhea or constipation), hematochezia, abdominal pain, distension, nausea, vomiting, weight loss, and anemia. Many of these symptoms overlap with pregnancy-related symptoms, often delaying diagnosis. Bernstein et al. reported that approximately 60% of colorectal cancers during pregnancy are diagnosed at stage III or higher.^4)^ Kocián et al. further noted that stage III or higher colorectal cancer accounts for 73.2% of all colorectal cases during pregnancy.^5)^

Treating colorectal cancer during pregnancy involves collaboration among surgeons, obstetricians, pediatricians, and anesthesiologists. Treatment decisions depend on the site and stage of the tumor, gestational age, and the health of the mother and fetus. FOLFOX and FOLFIRI are the mainstays of chemotherapy for colorectal cancer. However, as the 1st trimester represents the period of organogenesis, with a fetal malformation risk of up to 20%, chemotherapy is generally contraindicated.^6)^ Depending on the necessity of chemotherapy, pregnancy termination may be an option. We performed a PubMed review of chemotherapy for colorectal cancer during pregnancy^5,7–23)^ (**Table 1**). We found that chemotherapy is primarily administered in the 2nd and 3rd trimesters, with regimens such as FOLFOX and FOLFIRI considered relatively safe and feasible.^5,7–25)^ However, an association has been reported between the use of platinum-based chemotherapy during pregnancy and SGA (odds ratio 3.12, 95% CI 1.45–6.70). The proposed causes include direct effects due to the toxicity of the chemotherapy agents and indirect effects such as induction of vasculopathy and high maternal stress.^26)^ Furthermore, although chemotherapy after the early stages of pregnancy is not associated with an increased risk of fetal malformations, it is associated with an increased risk of stillbirth, intrauterine growth retardation, and fetal toxicity.^27)^ As depicted in **Table 1**, among the 32 reported cases, there were 2 stillbirths (6.3%), 11 premature births (34.4%), 8 SGA babies (25.0%), and 1 infant with a congenital malformation (3.1%). Similar to previous reports, the incidence of malformations after chemotherapy during pregnancy was the same as that in the general population. However, the incidences of stillbirth,^28)^ premature babies, and SGA babies were higher than those in the general population, necessitating a thorough explanation.

**Table 1:** Summary of reports of chemotherapy administered during pregnancy for colorectal cancer and our case

Previously reported cases	n = 32		Our case
Diagnosis		Diagnosis	
Average age (range)	32.6 (25–43) (n = 19)	Age	36
	Not reported (n = 13)		
Site of cancer, n (%)		Site of cancer	
Right side of colon	3 (9.4)		
Left side of colon	16 (50.0)		
Rectum	11 (34.4)		Rectum
Not reported	2 (6.3)		
Clinical stage, n (%)		Clinical stage	
Ⅰ and lower	0 (0)		
Ⅱ	3 (9.4)		Ⅱ
Ⅲ	9 (28.1)		
Ⅳ	20 (62.5)		
Chemotherapy during pregnancy		Chemotherapy during pregnancy	
Period of treatment, n (%)		Period of treatment	
1st trimester	0 (0)		
2nd and 3rd trimester	32 (100)		2nd and 3rd trimester
Regimen, n (%)		Regimen	
5-FU	8 (25.0)		
FOLFOX	19 (59.4)		FOLFOX
FOLFIRI	2 (6.3)		
FOLFOXIRI	2 (6.3)		
Other	1 (3.1)		
Obstetric and neonatal outcome		Obstetric and neonatal outcome	
Stillbirth, n (%)	2 (6.3)		
Premature, n (%)	11 (34.4)		Premature
Small for gestational age, n (%)	8 (25.0)		
Congenital malformation, n (%)	1 (3.1)		

Most previously reported cases were stage III or higher, with stage II cases, such as our case, being rare. Although the baby was premature, she was appropriate for gestational age and exhibited no obvious abnormalities, such as malformations.

Surgical treatment depends not only on the stage of pregnancy but also on the tumor site, while the timing of surgical treatment depends on the gestational age. Immediately after diagnosis, if the pregnancy is less than 20 weeks, the uterus is relatively small, making radical resection feasible. However, if the pregnancy exceeds 20 weeks, the uterus becomes enlarged, making radical resection for rectal cancer particularly difficult. Therefore, surgical treatment should be postponed for several weeks after delivery, allowing time for the uterus to involute.^29)^ In the present case, we experienced tearing of the uterus when it was lifted during the pelvic procedure. This tear was likely due to the fragility of the puerperal uterine tissues. To address this issue, we considered lifting the peritoneum of the Douglas pouch or using a uterine manipulator. In cases involving perforation or obstruction due to a tumor, emergency surgery becomes unavoidable. However, there are concerns about the potential effects of surgery and anesthesia on both the mother and fetus. Despite these concerns, advancements in modern technology have reduced maternal mortality rates. Moreover, even during early pregnancy, there has been no increase in congenital abnormalities in the fetus.^30)^ As presented in **Table 1**, our review revealed that approximately half of the patients underwent surgery during pregnancy; however, the potential effects of the surgery were not described.

The present case involved clinical stage II rectal cancer diagnosed at 22 weeks of gestation. Given that the pregnancy had surpassed 20 weeks, immediate surgery was not feasible, and it was deferred until after delivery, once the uterus had involuted. Considering the health of the fetus, a minimum gestational age of 28 weeks was necessary, requiring a delay of approximately 9 weeks before surgery. During this period, there was a risk of cancer progression and the baby being born prematurely. Ultimately, after weighing these risks against those of chemotherapy, the patient opted for chemotherapy during pregnancy.

To ensure the safety of both the mother and the fetus, neoadjuvant chemotherapy with FOLFOX was selected after thorough discussions with the patient, an obstetrician-gynecologist, a pediatrician, and the medical safety manager. After delivery, we also considered the potential risks associated with breastfeeding following the administration of anticancer drugs. Cancer chemotherapy is generally not recommended during breastfeeding.^29)^ Fluorouracil may be excreted into breast milk and has been associated with neutropenia in infants.^31)^ Additionally, oxaliplatin can raise platinum concentrations in breast milk, with accumulation during continuous administration. Although the exact effects on infants remain unclear, breastfeeding should be avoided.^32)^ In this case, to eliminate the risk of drug transfer through breast milk, breastfeeding was not initiated, considering the potential impact of postoperative adjuvant chemotherapy.

Various reports have been published on the prognosis of colorectal cancers during pregnancy. Chan et al. evaluated 42 cases of colon cancer during pregnancy and found that most patients succumbed within 1 year, with a mean survival of less than 5 months.^33)^ This poor prognosis can be attributed to delays in diagnosis, a decline in the cellular immunity of the mother owing to pregnancy, and an increase in pelvic blood flow, which increases the blood supply to the tumor and potentially promotes distant metastasis.^34)^ In contrast, a relatively recent report revealed that the 1-year survival rate for patients with all stages of colorectal cancer during pregnancy was 78.1%, whereas that for those with stage IV was 48.6%, which is comparable to the prognosis of the general population at that time.^5)^ In addition, a systematic review indicated that the median survival time of patients with colorectal cancer during pregnancy who underwent hepatectomy for metastasis was 42 (0–120) months.^25)^ The improved prognosis reported in recent studies is likely attributed to advances in diagnostic and therapeutic interventions during pregnancy. To further improve prognosis, early diagnosis and treatment interventions are essential. Notably, the importance of neoadjuvant chemotherapy during pregnancy is being increasingly recognized.

## CONCLUSIONS

Even in cases of clinical stage II colorectal cancer diagnosed during the 2nd trimester where immediate surgery is not feasible, neoadjuvant chemotherapy may be a viable treatment option. To establish standard treatment for similar cases, additional case accumulation and long-term follow-up data on children will be required in the future.

## ACKNOWLEDGMENTS

We would like to thank Editage (www.editage.jp) for editing the English language.

## DECLARATIONS

### Funding

This study received no funding.

### Authors’ contributions

HK wrote the manuscript.

DY assisted in the preparation of the manuscript.

ST, HT, RM, KM, HS1, KD, YS, TT, HS2, HM, JK, and NI critically revised the article and approved the final version for publication.

All authors have read and approved the final version of the manuscript.

### Availability of data and materials

Not applicable.

### Ethics approval and consent to participate

This work does not require ethical considerations or approval. Informed consent to participate in this study was obtained from the patient.

### Consent for publication

Informed consent was obtained from the patient for publication of this case report.

### Competing interests

The authors declare that they have no competing interests.
